# Metabolomic analysis reveals a protective effect of Fu-Fang-Jin-Qian-Chao herbal granules on oxalate-induced kidney injury

**DOI:** 10.1042/BSR20181833

**Published:** 2019-02-22

**Authors:** Wei Chen, Wen-Rui Liu, Jie-Bin Hou, Jia-Rong Ding, Zhong-Jiang Peng, Song-Yan Gao, Xin Dong, Jun-Hua Ma, Qi-Shan Lin, Jian-Rao Lu, Zhi-Yong Guo

**Affiliations:** 1 Changhai Hospital, Second Military Medical University, Shanghai 200433, China; 2 Department of Nephrology, Seventh People’s Hospital Affiliated to Shanghai University of Traditional Chinese Medicine, Shanghai 200137, China; 3 Department of Geriatric Nephrology, Chinese PLA General Hospital, Beijing 100853, China; 4 School of Pharmacy, Second Military Medical University, Shanghai 200433, China; 5 Guangxi Wantong Pharmaceutical Co., Ltd, 16 Dong Erli, South Zhongyao Road, Nanning, Guangxi 530003, China; 6 Proteomics/Mass Spec Facility, Center for Functional Genomics, University at Albany, Rensselaer, NY 12144, U.S.A.

**Keywords:** Fu-Fang-Jin-Qian-Chao, Kidney stone, mass spectrometry, metabolomics, Oxalate crystal

## Abstract

Nephrolithiasis is one of the world’s major public health burdens with a high incidence and a risk of persistent renal dysfunction. Fu-Fang-Jin-Qian-Chao granules (FFJQC), a traditional Chinese herb formula, is commonly used in treatment of nephrolithiasis. However, the therapeutic mechanism of FFJQC on kidney stone has still been a mystery. The objective of the present study is to explore the therapeutic mechanism of FFJQC on kidney injury and identify unique metabolomics patterns using a mouse model of kidney stone induced by a calcium oxalate (CaOx) deposition. Von Kossa staining and immuno-histopathological staining of osteopontin (OPN), cluster of differentiation 44 (CD44) and calbindin-D28k were conducted on renal sections. Biochemical analysis was performed on serum, urine, and kidney tissues. A metabolomics approach based on ultra-HPLC coupled with quadrupole-TOF-MS (UHPLC-Q-TOF/MS) was used for serum metabolic profiling. The immunohistopathological and biochemical analysis showed the therapeutic benefits of FFJQC. The expression levels of OPN and CD44 were decreased while calbindin-D28k increased after the CaOx injured mice were treated with FFJQC. In addition, total of 81 serum metabolites were identified to be associated with protective effects of FFJQC on CaOx crystal injured mice. Most of these metabolites were involved in purine, amino acid, membrane lipid and energy metabolism. Potential metabolite biomarkers were found for CaOx crystal-induced renal damage. Potential metabolite biomarkers of CaOx crystal-induced renal damage were found. FFJQC shows therapeutic benefits on CaOx crystal injured mice via regulation of multiple metabolic pathways including amino acids, purine, pyrimidine, glycerolipid, arachidonic acid (AA), sphingolipid, glycerophospholipid, and fatty acid.

## Background

Nephrolithiasis is a highly prevalent disease worldwide with rates ranging from 1 to 13% [1]. The prevalence is progressively rising due to various factors including metabolic dysfunction [2,3] and diet [4], jeopardizing public health and life quality of affected individuals. Formation of kidney stones can result in the loss of function of kidney and increase the risk of chronic kidney diseases [5]. In addition, several epidemiologic studies have also found that the occurrence of a kidney stone is associated with a higher risk of cardiovascular diseases [6], such as coronary heart disease and stroke. Therefore, basic research is needed for the mechanism, prevention, and treatment of kidney stones.

Kidney crystals are early forms of kidney stones [7]. At an early stage of kidney stone, the crystals are deposited in renal tubules and cause to expand the lumen of the tubules. The cells injured to a degree of complete cell necrosis which is accompanied by interstitial inflammation and fibrosis. In advanced stage, glomerular sclerosis, tubular atrophy, and interstitial fibrosis were found [8].

Nephrolithiasis requires formation of crystals followed by their retention and accumulation in the kidney, while crystals retention in kidney depend on the expression of cluster of differentiation 44 (CD44) and osteopontin (OPN) by injured/regenerating tubular cells [9,10]. Calbindin-D28k is a cytosolic calcium binding protein located in the distal convoluted tubule and plays an important role in active calcium transport in the kidney. Some other calcium transport proteins including calcium-sensing receptor (CaSR) have been reported to be associated with hypercalciuria and nephrolithiasis [11].

The oxalate crystals of the renal tubules can interact with renal tubular epithelial cells. Then, the oxalate crystals adhere to the epithelial cells. Final deposition of crystals leads to the formation of kidney stones [12]. Calcium oxalate (CaOx) is the major component (approximately 80%) of kidney crystals or stones [13]. Oxidative stress and inflammation play significant roles throughout the entire process of CaOx stone formation. With the aggregation and growth of CaOx crystal, the inflammation injury and crystal-induced reactive oxygen species (ROS) activation could in turn promote the further formation of stones [14,15]. Intervention of CaOx crystal nephropathy is considered the key for prevention and treatment of nephrolithiasis. Inhibition of oxidative stress has been demonstrated to alleviate CaOx-induced injury and prevent renal stone formation [16].

Traditional Chinese herb formula (Chinese Drug Standards: 2016B00026), Fu-Fang-Jin-Qian-Chao (FFJQC) granules [17], has been used for the treatment of urolithiasis and urinary infection [18]. It consists of *Desmodium styracifolium* (Osbeck.) Merr., *Plantago asiatica* L., *Pyrrosia calvata* (Baker) Ching, and *Zea mays* Linn. (Table 1). The effective component of FFJQC is *D. styracifolium* (DS), a popular plant widely distributed in southern China. Previous studies demonstrated that it could be used for removal of renal CaOx depositions [19,20]. However, little is known about the mechanism of its anti-urolithiasis effect. The therapeutic mechanism of FFJQC is mystery in the aspect of metabolomic pathways. In the present study, we used a serum metabolomics-based approach and renal immunological staining techniques to examine the therapeutic effects of FFJQC in a mouse model of urolithiasis, which is characterized by renal CaOx deposition.

**Table 1:** Typical composition of FFJQC granules

Botanical plant name	Family	Part used	Chinese name	Ratio
*Desmodium styracifolium* (Osbeck.) Merr.	Leguminosae	Aerial part	Guang Jin Qian Cao	4
*Plantago asiatica* L.	Plantaginaceae	Herbs	Che Qian Cao	2
*Pyrrosia calvata* (Baker) Ching	Polypodiaceae	Herbs	Guang Shi Wei	2
*Zea mays* Linn.	Gramineae	Style and stigma	Yu Mi Xu	1

## Materials and methods

### Chemicals and reagents

Glyoxylic acid was obtained from Tokyo Chemical Industry (TCI, Tokyo, Japan). Chromatographic grade methanol and acetonitrile were purchased from Merck KGaA (Darmstadt, Germany). Formic acid was obtained from Fluka (Buchs, Switzerland). Ultrapure water was prepared using a Milli-Q water purification system (Millipore Corp., MA, U.S.A.). All other chemicals were of analytical grade.

### Assessment of the quality of FFJQC

The reference standards of FFJQC, including mangiferin [21] and schaftoside [22], were precisely weighed and placed into a 50-ml bottle. Methanol (50% v/v) was used to dissolve the compounds. The granules of the FFJQC were provided by Guangxi Wantong Pharmaceutical Co., Ltd (Guangxi, China) and ground to powder. A total of 2 g powdered sample was extracted ultrasonically in 25 ml of 50% methanol (v/v) for 10 min. The mixture was then filtered and the filtrate (10 ml) was evaporated to dryness. The residue was re-dissolved with 5 ml mobile phase (0.2% phosphoric acid). The solution was filtered through a 0.45-μm filter membrane before HPLC analysis. Two samples of FFJQC were used from each batch.

HPLC was performed on a 1525 HPLC system (Waters, MA, U.S.A.) to identify the main chemical constituents in FFJQC to assess their quality. The separation of the FFJQC samples and the standard sample was carried out on a Wondasil C18 column. The flow rate was 1 ml/min with an injection volume of 20 μl. The wavelength was set at 350 nm. The mobile phase consisted of mobile phase A (0.2% phosphoric acid in water) and mobile phase B (acetonitrile). The gradient elution program was as follows: 7–13.5% B from 0 to 8 min, 13.5–15% B from 8 to 26 min, 15–27% B from 26 to 54 min, and 27–7% B from 54 to 55 min.

### Animal experiment and sample collection

Male C57BL/6 mice (8 weeks old) were purchased from the Shanghai SLAC Lab Animal Co, Ltd. (Shanghai, China). After conditional housing for 1 week, these mice were randomly divided into four experimental groups including saline, oxalate, FFJQC, and FFJQC only. There were six mice in each group. To establish the crystal renal injury model, the mice were intraperitoneally (i.p.) injected with glyoxylate at a dosage of 100 mg/kg once daily for 6 days according to previous experimental methods [23,24]. FFJQC was dissolved in saline solution to obtain a concentration of 270 mg/ml and the mice given FFJQC were intragastrically (i.g.) administrated the FFJQC at a dose of 2.7 g/kg body weight. The mice in oxalate and FFJQC groups were i.p. injected with glyoxylate once daily for 6 days, while saline and FFJQC only groups were i.p. injected with a similar volume of normal saline. Four hours after the glyoxylate/saline i.p injection, mice in FFJQC and FFJQC only groups were i.g. administrated the FFJQC once daily for 6 days while the saline and oxalate groups were i.g. administrated a similar volume of normal saline. Twenty-four-hour urine was collected on day 6 after the administration of glyoxylate. At the end of the experiment, the mice were all anesthetized with sodium thiopental. The blood samples were collected by retro-orbital puncture. After clotting at 4°C for 2 h, the venous blood was centrifuged at 4000 rpm (1163 ***g***-force) for 5 min. The supernatant was taken as serum sample and stored at −80°C prior to analysis. After *in situ* cardio-perfusion, the right kidneys were removed immediately and stored for biochemistry detection at −80°C. The left kidneys were fixed in 10% buffered formalin for pathological analysis. All the animal studies were performed in accordance with the National Institutes of Health (NIH) guideline for the Care and Use of Laboratory Animals. The experimental procedures were approved by the Ethical Committee for the Experimental Use of Animals at the Second Military Medical University (Shanghai, China).

### Histopathological and biochemistry analyses

Kidney samples were further paraffin-embedded and sectioned at a thickness of 3 μm for light-microscopic examination. The sections stained with Hematoxylin–Eosin (HE) were reviewed by an independent pathologist, and 20 images of vision in 400× were randomly selected from each mouse. The severities of tubular changes in each image were examined and classified as 3 point, severe; 2 point, moderate; 1 point, mild; 0 point, negative according to the Carraway’s Scoring [25].

The sections stained with Von Kossa underwent deparaffinization and hydration using a series of dilutions of xylene and alcohol, followed by staining using the Von Kossa kit (Jiemei Gene, Shanghai, China) and subsequent Eosin counterstaining (Beyotime Institute of Biotechnology, Jiangsu, China). The stained slices were then assessed using microscopy (Nikon Eclipse 50i; Nikon Corporation, Tokyo, Japan) for the distribution of CaOx crystals, characterized by black CaOx crystal deposits. The number of crystals in a total cross-sectional tissue area was determined using Adobe Photoshop software version 7.0 (Adobe Systems, Inc., CA, U.S.A.) in 20 randomly selected fields (magnification: ×200).

Immunohistochemical staining was performed according to the manufacturer’s instructions of a commercial kit (SA1020, Boster, Hubei, China). Paraffin sections were high-pressure treated for 2 min and blocked with 0.5% H_2_O_2_ in methanol for 15 min, washed in 0.01 M PBS with Tween-20 (PBST) and further treated with non-fat milk in PBS for 30 min at room temperature. The slides were then incubated overnight at 4°C with primary antibodies against OPN (1:50) and CD44 (1:100), followed by incubation with the secondary antibody. The positive staining of OPN and CD44 were measured as the ratio of integral optical density/field of kidney cross-sections, using Image-Pro Plus software version 6.0. A mouse monoclonal antibody against OPN (sc-73631) was purchased from Santa Cruz Biotechnology, Inc. (CA, U.S.A.), a rabbit polyclonal antibody against CD44 (15675-1-AP) was purchased from Proteintech Group (CA, U.S.A.), a mouse polyclonal antibody calbindin-D28K (PAG438Mu01) was purchased from Cloud-Clone Corp. (Wuhan, China). The appropriate secondary antibodies for immunohistochemical staining were purchased from Santa Cruz Biotechnology, Inc, and that for the immunofluorescence was purchased from Jackson Immuno Research Laboratories (NJ, U.S.A.). Semi-quantitative analysis of OPN, CD44, and calbindin-D28k were calculated by Image-Pro Plus 6.0 (Media Cybernetics, MD, U.S.A.). Twenty views (magnifcation: ×200) were gathered from each group.

The levels of calcium and creatinine in urine, the levels of serum creatinine (Scr) and blood urea nitrogen (BUN) were measured by a Cobas® C311 Autoanalyzer (Roche Diagnostics, Indianapolis, IN, U.S.A.). The calcium content of kidney tissue was measured by using a colorimetric assay kit (Nanjing Jiancheng, Jiangsu, China), according to the manufacturer’s instructions. A portion of the kidney tissue was homogenized with normal saline, and then centrifuged at 2500 rpm (454 ***g***-force) for 10 min at 4°C. The resultant supernatant was equilibrated with saline and the levels of proteins in the tissue homogenates was detected by a BCA protein assay kit of Nanjing Jiancheng (Jiangsu, China). The levels of catalase (CAT), superoxide dismutase (SOD), glutathione reductase (GSH), and malondialdehyde (MDA) were determined by commercial kits of Nanjing Jiancheng (Jiangsu, China). The measurements were performed strictly following the manufacturer’s instructions.

### Serum sample preparation

Prior to the analysis, a 100-μl aliquot of serum sample was thawed at 4°C followed by the addition of 300 μl of acetonitrile to precipitate the proteins. The resulting solution mixture was spun at 13000 rpm (12281 ***g***-force) for 15 min at 4°C. The supernatant (150 μl) was transferred to the sample vial for ultra HPLC (UHPLC)–MS analysis.ultra-HPLC coupled with quadrupole-TOF-MS.

### Ultra-HPLC coupled with quadrupole-TOF-MS profiling analysis

Ultra-HPLC coupled with quadrupole-TOF-MS (UHPLC-Q-TOF/MS) analysis was performed on an Agilent 1290 Infinity LC system equipped with Agilent 6530 Accurate Mass Quadrupole Time-of-Flight mass spectrometer (Agilent Technologies Inc., CA, U.S.A.). Chromatographic separations were performed at 40°C on an HSS T3 column (2.1 mm I.D. × 100 mm, 1.8 μm, Waters, MA, U.S.A.). The mobile phase consisted of 0.1% formic acid (A) and acetonitrile modified with 0.1% formic acid (B). The optimized UPLC elution condition was: 0–2 min, 5% B; 2–10 min, 5–15% B; 10–14 min, 15–30% B; 14–17 min, 30–95% B; 17–19 min, 95% B, and the post time was 6 min for equilibrating the system. The flow rate was set to 0.4 ml/min and the injection volume was 2 μl. The autosampler was maintained at 4°C. An ESI source was operated in both positive and negative modes. The ESI conditions involved nitrogen drying gas at a flow rate of 11 l/min and a temperature of 350°C, a nebulizer gas (nitrogen) pressure of 45 psi, fragmentor voltage of 120 V, skimmer voltage of 60 V, and a capillary voltage of 3500 V in a positive mode and 4000 V in a negative mode. The potential metabolites were further analyzed by MS/MS, and the collision energy was set from 10 to 50 eV.

### Data process and statistical analysis

The UHPLC–MS raw data were converted into a common data format (mzdata) files using Agilent MassHunter Qualitative software. The isotope interferences were excluded and the threshold was set to 0.1%.

The program XCMS (http://metlin.scripps.edu/download/) was applied for peak alignment of the data in the time domain, extraction of the peak intensities, and automatic integration. After filtering the ions based on the 80% rule, the detected ions in each sample were normalized to the sum of the peak area to acquire the relative intensity of metabolites. Then, the 3D data matrix including the sample names, retention times, m/z pairs, and normalized ion intensities were imported into the SIMCA-P program (version 11.0, Umetrics, Umea, Sweden) for multivariate statistical analysis after mean-centering and Pareto scaling. Principal component analysis (PCA) and partial least squares discriminant analysis (PLS-DA) were employed. The permutation tests were done to evaluate the quality of the models. The variable importance in the projection (VIP) values were generated and represented the contribution to the intergroup discrimination of each metabolite ion. Metabolite ions with VIP values greater than 1.0 were selected for further analysis.

The statistical significance of mean values were tested using the one-way ANOVA and the post hoc Tukey’s test through SPSS 19.0 program (IBM, NY, U.S.A.). The differences were considered significant when a *P*-value was less than 0.05. The heatmap of the different metabolites was performed on the MetaboAnalyst platform (http://www.metaboanalyst.ca).

### Identification of the potential biomarkers

It is important and pretty challenging to identify different metabolites in a complex biological system. First, we found the ions based on the extracted ion chromatogram. Second, we inputted the accurate molecular mass of the ions into online databases to search for metabolite candidates including Metlin (http://metlin.scripps.edu/), Human Metabolome Database (http://www.hmdb.ca/), and Mass Bank (http://www.massbank.jp/). Third, we compared the MS/MS spectra with the MS/MS information from databases to verify the structure of the putative metabolites. Finally, the metabolites were validated using standard synthetic samples based on the retention time and information on collision induced dissociation (CID) fragments, i.e., MS/MS spectrum.

## Results

### HPLC profile of FFJQC

**Figure 1:**
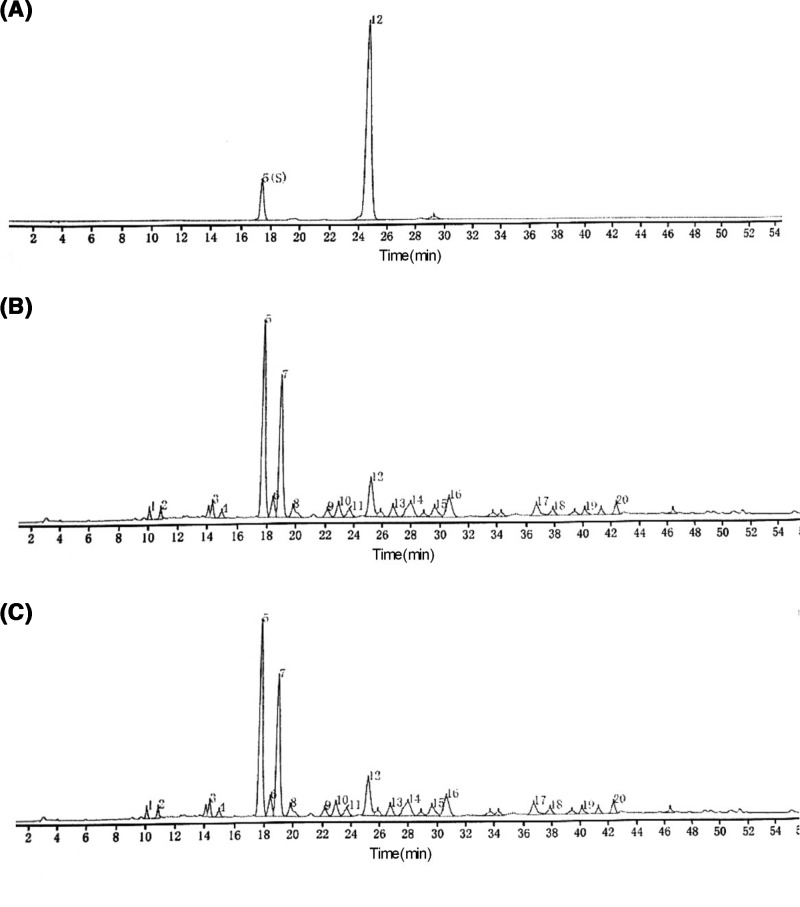
High performance liquid chromatograms of FFJQC and the reference standards (**A**) Chromatogram of standards with mangiferin(5) and schaftoside(12). (**B**,**C**) Chromatograms of two batches of FFJQC provided by Guangxi Wantong Pharmaceutical Co., Ltd. (180627/1 and 180705/1).

The representative HPLC chromatograms of FFJQC and their reference standards were provided in Figure 1. Based on the comparisons with the retention times of standard compounds, one major component of FFJQC was identified as mangiferin, while the other was uniquely identified as schaftoside. The result demonstrated that all batches of FFJQC meet the Chinese Pharmacopoeia standard and can be used in the following set of experiments.

### Histopathological and biochemistry tests

**Figure 2:**
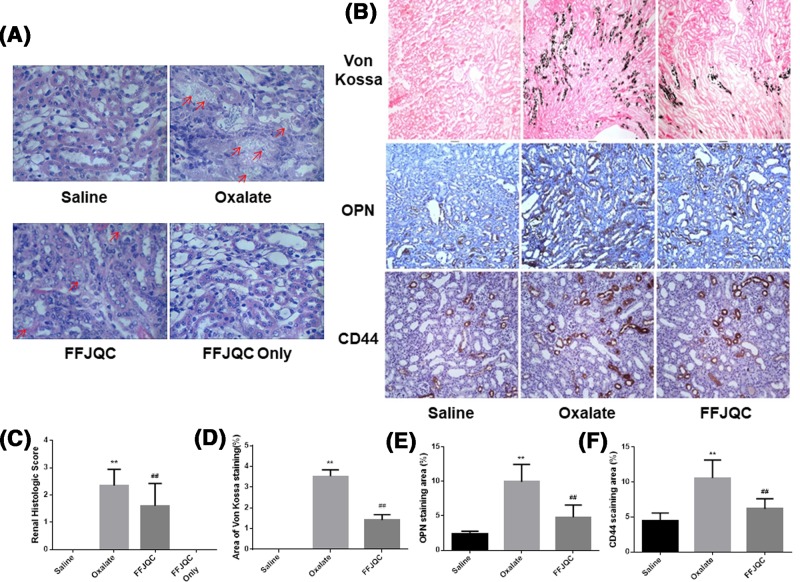
Histological analysis of mouse model induced by oxalate (**A**) Representative images of HE staining of kidney cortex and medulla junction. (**B**) Representative photomicrographs of Von Kossa staining of calcium deposition and immunostaining of OPN and CD44 expression. The crystal deposition was clearly increased in the oxalate mouse group. After the FFJQC treatment, the crystal deposition was decreased significantly in this group. The protein expression levels of OPN and CD44 were increased in the oxalate group, then their levels were decreased significantly after the FFJQC treatment. (**C**) Tubulointerstitial damage were assessed semiquantitatively according to Carraway’s scoring from every 20 random views. (**D**) Semi-quantitative analysis of calcium deposition in the area of positive staining from 20 random views. (**E**,**F**) Semi-quantitative analysis of OPN and CD44 expression in each group from 20 random immunohistochemical views. (C) **P<0.0001 Oxalate group compared with Saline group, ^##^*P*=0.002 FFJQC group compared with Oxalate group. (D) **P<0.0001 Oxalate group compared with Saline group, ^##^*P*<0.0001 FFJQC group compared with Oxalate group. (E) **P<0.0001 Oxalate group compared with Saline group, ^##^*P*<0.0001 FFJQC group compared with Oxalate group. (F) **P<0.0001 Oxalate group compared with Saline group, ^##^*P*<0.0001 FFJQC group compared with Oxalate group.

Kidney stone formation, immunohistochemical images of related protein expression results are demonstrated in Figure 2. Based on the HE staining, the crystals are deposited in the renal tubules. The renal tubule epithelium injury score in the Oxalate group was significantly higher than that of the Saline group. However, the injury score was reversed in the FFJQC group (Figure 2A). In Von Kossa staining, the CaOx crystals generated in the tubular lumens located between the renal medulla and the cortex, were obviously reduced by FFJQC (Figure 2B). The staining for OPN and its receptor CD44 suggested that the protein expression levels of OPN and CD44 in the tubular cells were higher in the oxalate group than that of the saline group. Furthermore, their expressions were significantly lower in the FFJQC group as compared with those of the oxalate group (Figure 2B).

**Figure 3:**
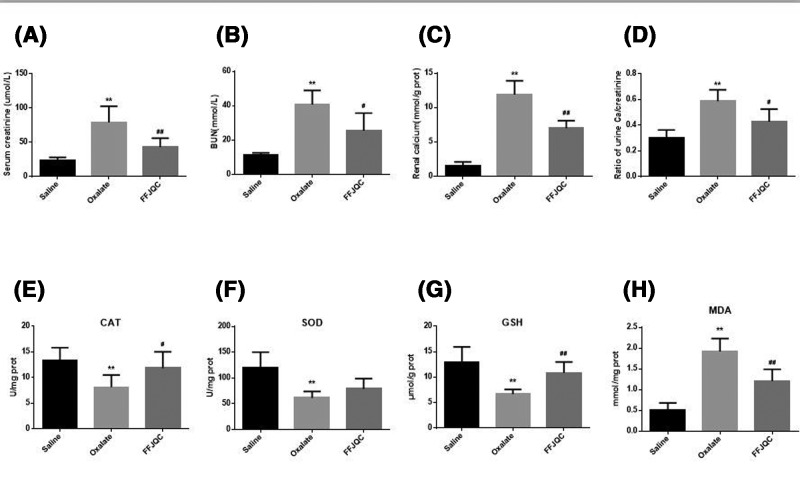
Biochemical analysis of mouse model induced by oxalate Levels of Scr (**A**), BUN (**B**), renal calcium (**C**), and urine Ca/creatinine (**D**) were determined (**P<0.01 oxalate group compared with saline group, ^#^*P*<0.05, ^##^*P*<0.01 FFJQC group compared with oxalate group). All data are expressed as the mean ± S.D. (**E**) Concentration of CAT in the kidney, (**F**) concentration of SOD in the kidney, (**G**) concentration of GSH in the kidney, (**H**) concentration of MDA in the kidney. (A) **P=0.0002 Oxalate group compared with Saline group, ^##^*P*=0.0081 FFJQC group compared with Oxalate group; (B) **P<0.0001 Oxalate group compared with Saline group, ^#^*P*=0.0177 FFJQC group compared with Oxalate group; (C) **P<0.0001 Oxalate group compared with Saline group,^ ##^*P*=0.0004 FFJQC group compared with Oxalate group. (D) **P<0.0001 Oxalate group compared with Saline group, ^##^*P*=0.0139 FFJQC group compared with Oxalate group; (E) **P=0.0005 Oxalate group compared with Saline group, ^##^*P*=0.0447 FFJQC group compared with Oxalate group; (F) **P=0.0014 Oxalate group compared with Saline group, ^##^*P*=0.0947 FFJQC group compared with Oxalate group; (G) **P=0.0007 Oxalate group compared with Saline group, ^##^*P*=0.0014 FFJQC group compared with Oxalate group; (H) **P<0.0001 Oxalate group compared with Saline group, ^##^*P*=0.0019 FFJQC group compared with Oxalate group.

The levels of BUN and Scr were higher in the oxalate group, as compared with the control group. The level of BUN and Scr in the FFJQC group decreased signifcantly (Figure 3A,B). The concentration of calcium in renal tissue increased significantly after the mice were administered glyoxylate. Interestingly, it was decreased significantly in the FFJQC group (Figure 3C). The significantly higher ratio of urine calcium/creatinine in the Oxalate group compared with the Saline group could be reversed by FFJQC (Figure 3D).

The levels or activities of CAT, SOD, GSH, and MDA in renal tissues were determined to assist the FFJQC in protecting renal tissue from oxidative damage (Figure 3E–H). The level of MDA increased significantly (*P*<0.01), and the activity of CAT and SOD and the GSH level decreased significantly (*P*<0.01) in the Oxalate group. In the FFJQC group, a significant reduction in MDA level (*P*<0.01) was observed, and a significant increase in CAT (*P*<0.05) activity and GSH level (*P*<0.01) was observed. However, the level of SOD did not change significantly after the treatment of FFJQC.

**Figure 4:**
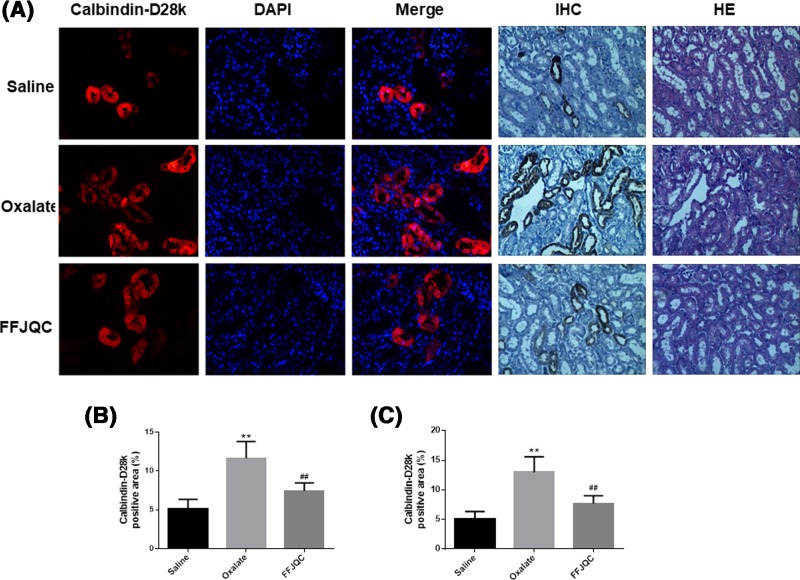
Representative immunofluorescent(IF) and immunohistochemical (IHC) staining of calbindin-D28k at the corticomedullary junction in kidneys The calbindin-D28k protein were labeled with red fluorescence and the nuclei were labeled with blue fluorescence. The images were taken under fluorescence microscope-Magnification: 400×, optical microscope-Magnification: 200×. (**A**) The calbindin-D28k expression level decreased in the oxalate mouse group. After the FFJQC treatment, the calbindin-D28k expression level increased significantly. (**B**) Quantitation of calbindin-D28k+ area in IF, **P<0.001 Oxalate group compared with Saline group, ^##^*P*=0.0015 FFJQC group compared with Oxalate group. (**C**) Quantitation of calbindin-D28k+ area in IHC, **P<0.001 Oxalate group compared with Saline group, ^##^*P*=0.0021 FFJQC group compared with Oxalate group. Twenty specimens were used while calculating the calbindin-positive area.

Based on the immunofluorescent and immunohistochemical image (Figure 4), calbindin-D28k was expressed in the cytoplasm of tubular cells in kidney tissues as shown by co-staining with DAPI, demonstrating that calbindin-D28k expression level decreased in the oxalate mouse group. However, it was reversed and up-regulated in the tubular cells when the mice were treated with FFJQC, indicating a renoprotective effect of FFJQC.

### Metabolic profiling analysis of serum

**Figure 5:**
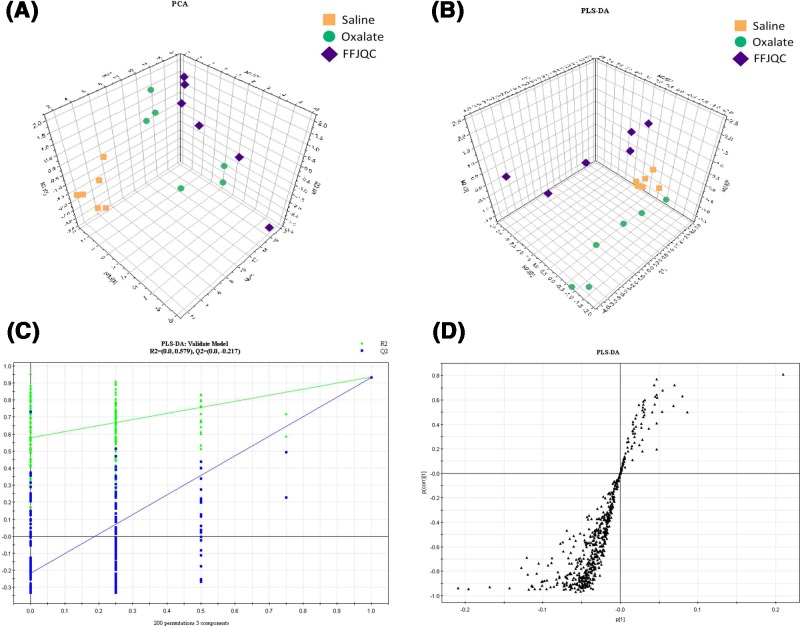
Plots of multivariate statistical analysis based on the serum metabolites in ESI positive ion mode (**A**) PCA scores plot of saline, oxalate, and FFJQC groups. (**B**) PLS-DA scores plot of saline, oxalate, and FFJQC groups. (**C**) Validation plot obtained from permutation tests. (**D**) S-plot of the PLS-DA model.

The serum metabolic profiling analysis was performed as described in the ‘Materials and methods’ section. Typical total ion chromatograms (TICs) obtained from the ESI positive and ESI negative are shown in Supplementary Figures S1 and S2. The scores plot of PCA, an unsupervised multivariate data analysis technique, displayed that there were no outliers in the data. The plot revealed obvious differences from the three groups in both positive (Figure 5A) and negative ion mode (Supplementary Figure S3A). Then, we performed unsupervised PLS-DA to further differentiate the metabolite features for potential markers of metabolites. As shown by the scores plot (Figure 5B and Supplementary Figure S3B), the oxalate and the FFJQC groups were clearly separated from the normal control, which suggested that metabolic perturbation had occurred in the three groups. Permutation testing (Figure 5C and Supplementary Figure S3C) was performed on the quality of the model and indicated that the model was not over-fitted. The S-plot (Figure 5D and Supplementary Figure S3D) can be used to show the differential metabolite profiles. The ions which are far away from the origin contribute significantly to the clustering of the three groups and may be selected as potential biomarkers.

### Evaluation and biomarkers of the therapeutic mechanism of FFJQC on oxalate-induced kidney injury

**Figure 6:**
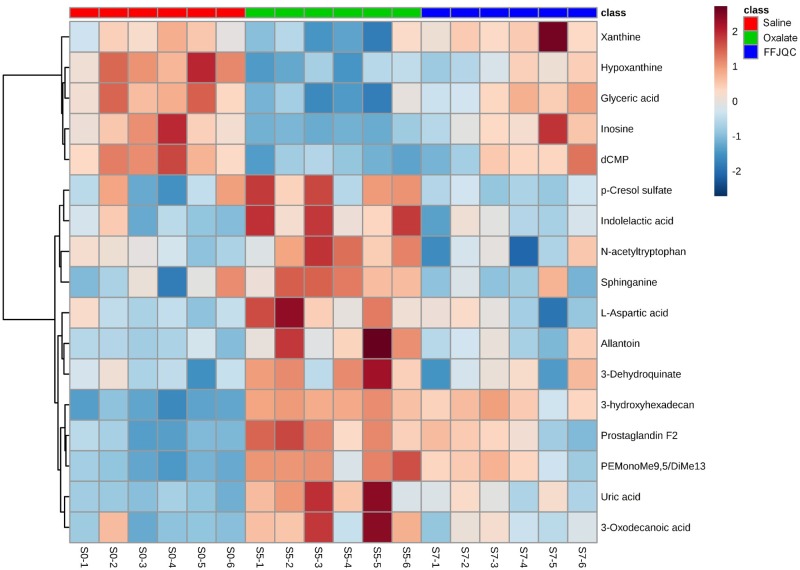
Heatmap generated from the relative levels of key metabolite biomarkers in mouse serum from groups of saline, oxalate, and FFJQC treated

The metabolites with significant differences between the saline and the oxalate group were identified. The trends of 81 identified metabolites were reversed by the FFJQC intervention (listed in Supplementary Table S1). The results indicated that the metabolic patterns in the serum of mice with glyoxylate-induced renal injury were changed to the normal levels after the mice were treated with FFJQC. Amongst the 81 reversed metabolites identified, 17 metabolites were rebound significantly in the FFJQC treated group as compared with the oxalate group. They could be used as key potential biomarkers to monitor the apparent protective effects of FFJQC against crystal-induced renal injury. Detailed changes and related pathways of the 17 key biomarkers are shown in Table 2. To visualize and characterize the benefit of the FFJQC treatment effectively, the heatmap representing the intensity levels of the 17 key metabolite biomarkers in the different groups are used (Figure 6).

**Table 2:** Biomarker candidates of glyoxylate-induced crystal kidney injury and their metabolic pathways

Number	RT (min)	m/z	Ion	Formula	Identification	Oxalate/Saline			FFJQC/Oxalate			Related pathway
						*P*-value	FDR	Fold change	P value	FDR	Fold change	
1	0.68	132.029	[M-H]^−^	C4H7NO4	l-Aspartic acid	0.016	0.023	1.44	0.010	0.024	0.68	Alanine, aspartate and glutamate metabolism
2	0.70	157.036	[M-H]^−^	C4H6N4O3	Allantoin	0.004	0.008	1.48	0.018	0.028	0.73	Purine metabolism
3	0.99	135.031	[M-H]^−^	C5H4N4O	Hypoxanthine	<0.001	<0.002	0.16	0.002	0.034	4.84	Purine metabolism
		137.046	[M+H]^+^									
4	0.99	153.041	[M+H]^+^	C5H4N4O2	Xanthine	0.030	0.002	0.42	0.003	0.017	2.92	
5	0.99	169.036	[M+H]^+^	C5H4N4O3	Uric acid	<0.001	<0.002	3.72	0.009	0.026	0.54	Purine metabolism
6	0.99	151.023	[M+FA-H]^−^	C3H6O4	Glyceric acid	<0.001	<0.002	0.27		0.034	3.02	Glycerolipid metabolism
7	1.08	189.041	[M-H]^−^	C7H10O6	3-Dehydroquinate	0.023	0.028	1.34	0.045	0.048	0.77	Phenylalanine, tyrosine and tryptophan biosynthesis
8	1.13	267.075	[M-H]^−^	C10H12N4O5	Inosine	<0.001	<0.002	0.03	0.008	0.027	21.04	Purine metabolism
		269.088	[M+H]^+^									
9	1.14	352.053	[M+FA-H]^−^	C9H14N3O7P	dCMP	<0.001	<0.002	0.23	0.024	0.034	2.98	Pyrimidine metabolism
10	5.81	187.008	[M-H]^−^	C7H8O4S	*p*-Cresol sulphate	0.048	0.048	1.89	0.017	0.029	0.42	Degradation of aromatic compounds
11	6.27	206.081	[M+H]^+^	C11H11NO3	Indolelactic acid	0.004	0.008	1.58	0.007	0.03	0.66	Tryptophan metabolism
		204.067	[M-H]^−^									
12	6.33	247.107	[M+H]^+^	C13H14N2O3	N-acetyltryptophan	0.017	0.022	1.71	0.034	0.041	0.63	Tryptophan metabolism
		245.094	[M-H]^−^									
13	9.19	185.117	[M-H]^−^	C10H18O3	3-Oxodecanoic acid	0.004	0.008	2.51	0.029	0.038	0.54	Pyrimidine metabolism
14	10.65	353.235	[M-H]^−^	C20H34O5	Prostaglandin F2a	<0.001	<0.002	3.95	0.015	0.032	0.61	Arachidonic acid metabolism
15	11.52	302.305	[M+H]^+^	C18H39NO2	Sphinganine	0.029	0.033	1.67	0.016	0.03	0.56	Sphingolipid metabolism
16	13.72	864.578	[M-H]^−^	C48H84NO10P	PE (MonoMe (9,5)/DiMe (13,5))	<0.001	<0.002	2.85	0.037	0.042	0.74	Glycerophospholipid metabolism
17	15.66	271.229	[M-H]^−^	C16H32O3	3-hydroxyhexadecanoic acid	<0.001	<0.002	2.49	0.048	0.048	0.87	Fatty acid metabolism

Abbreviations: FDR, false discovery rate; PE, phosphatidylethanolamine.

**Figure 7:**
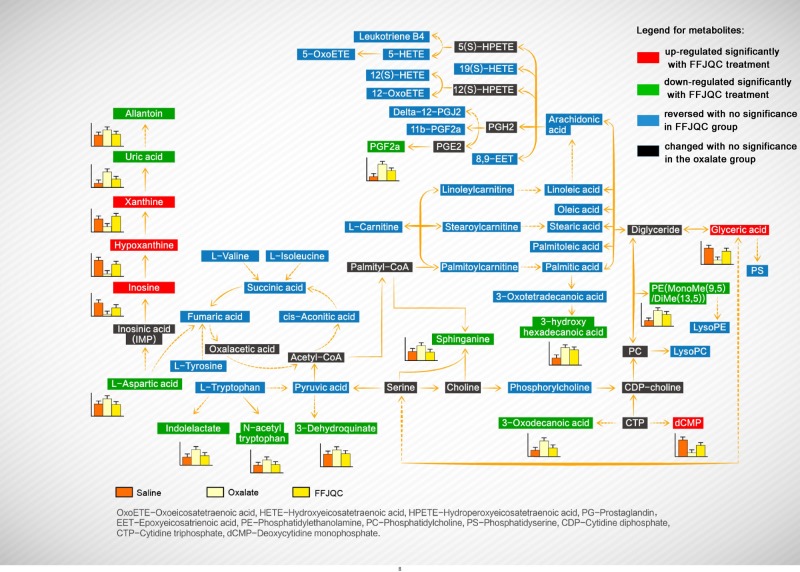
Proposed model of metabolic pathway networks resulting from the glyoxylate-induced crystal kidney injury and FFJQC treatment

Amongst the 81 reversed metabolites, 17 key metabolites were identified as potential biomarkers for the treatment of FFJQC in crystal renal injury. By searching the KEGG pathway database (http://www.genome.jp/kegg/), we constructed their metabolic network (Figure 7). The metabolic network shows that the protective effect of FFJQC is related to the perturbation of purine metabolism, amino acid metabolism, membrane lipids metabolism, and energy metabolism. Interestingly, the 17 key identified biomarkers (labeled in red or green) with significant rebound in the FFJQC treated group are mostly involved in purine metabolism, indicating that this metabolism pathway may play a key role in mediating the protective effect of FFJQC on crystal-induced kidney injury.

## Discussion

### Histopathological and biochemistry results

The protein expression levels of OPN and its cell surface receptor CD44 were increased in the oxalate group, which is consistent with previous findings [9]. It has been reported that knockdown of OPN resulted in significant decrease in the amount of CaOx crystal attachment to tubular cells. The expression of OPN and CD44 by injured tubular cells may play a role in the retention of crystals in the kidney [26,27]. OPN, a secreted glycoprotein, acts as a proinflammatory cytokine and activator of T lymphocytes. Blockade of the OPN signaling can reduce the levels of inflammation and renal injury caused by ischemia [28] and nephrotoxicity [29]. Our studies suggest that FFJQC could be used to decrease the crystallization and prevent the renal injury via reducing the expression of OPN and CD44 in kidney.

Testosterone is found to increase urinary calcium excretion with decreased expression of intracellular calbindin-D28K transporters [30], implying a correlation of calbindin-D28K with urinary calcium re-absorption. Calbindin-D28k was thought to play an important role in the calcium conservation [31]. In this study, one of the protective mechanisms of FFJQC treatment was probably related to the reversed expression of calbindin-D28k in the tubular cytoplasm.

### Induced purine metabolism perturbation

Purine metabolism was identified as a key metabolic pathway for a protective effect of FFJQC in crystal renal injury. Amongst the purine biomarkers in the FFJQC group, inosine, hypoxanthine, and xanthine are up-regulated as beneficial biomarkers while uric acid and allantoin are down-regulated as the adverse ones.

Allantoin is the end product of oxidation of uric acid through purine catabolism. After birth, it is a predominant carrier by which nitrogenous waste is excreted in the urine of many animals including rats. The increased level of allantoin was found in the renal tissue with ischemia injury in rat kidney transplants [32]. In human beings, however, the metabolic pathway for the conversion of uric acid into allantoin does not exist, so the former is mainly excreted. Due to the lack of metabolic enzyme of uric acid to allantoin in human, allantoin is produced only through non-enzymatic processes with ROS. Therefore, allantoin could be used to determine the oxidative stress in humans [33]. More clinical studies would be needed to determine the significance of allantoin in human. Clinically, serum uric acid is usually accumulated due to the reduced excretion by the crystal injured kidney. Uric acid is produced from xanthine and hypoxanthine, which in turn are produced from other purines. This metabolic reaction can be catalyzed by xanthine dehydrogenase. Xanthine dehydrogenase can be converted into xanthine oxidase (XO) in some special situations as alternative catalyzing enzyme for this reaction.

XO, an enzyme that regulates ROS, plays an important role in the catabolism of purines in humans. The significant decrease in xanthine in the oxalate group may be related to the activation of XO. Previous study showed that increased expression or activity of XO in the kidney induced by high sodium could indicate exaggerated oxidative stress. This could lead to massive crystal formation in the hyperoxaluric kidney [34]. The highly activated XO increased the level of intracellular ROS, which caused renal injury by direct oxidative damage to renal cells [35]. Allopurinol, a XO inhibitor, can attenuate the response of oxidative stress in renal ischemia–reperfusion injury, as being shown by a decrease in MDA level and apoptotic renal tubular cells [36]. The use of febuxostat, another XO inhibitor, can inhibit renal interstitial inflammation and fibrosis in unilateral ureteral obstructive nephropathy [37]. As an oxidant molecule, the increased XO in serum may reflect the systemic condition of oxidative stress. CaOx crystal was indicated to induce the oxidative stress of kidney. The modulation of oxidative stress could ameliorate the crystal-induced renal injury [38,39]. These were verified by detecting ROS in the kidney tissues. Therefore, the function of FFJQC in the present study are reasoned to reduce the systemic damage resulted from kidney crystals by relieving the oxidative stress reaction of the whole body.

As a former metabolite of purine, inosine is very important in kidney injury. Inosine is found to have a protective effect on the morphology of the rat nephron with warm ischemia [40]. The cytoprotection elicited by inosine in a model of renal ischemia involved both interactions with cell surface adenosine receptors on renal tubular epithelial cells and intracellular metabolism and conversion of adenosine into ATP [41]. A previous similar metabolomic research on mouse crystal-induced kidney injury has indicated that Orthosiphon stamineus, one of ancient traditional Chinese medicines, can cure disease by intervening in some dominating urinary metabolic pathways including purine metabolism [42]. Other serum metabonomics studies also showed the importance of purine metabolism in chronic kidney disease represented by hypoxanthine, inosine, and allantoin. *p*-Cresol sulphate, one of the uremic toxins, was demonstrated as a key reversed biomarker, which is consistent with our results.

N-Acetyl-N-nitroso-tryptophan (NANT) is an excellent NO donor for cells in culture. Uric acid may act as an effective transporter of nitric oxide, which receives a nitroso group from NANT, and passes it to the thiol group of N-acetylcysteine resulting in the production of N-acetyl-S-nitrosocysteine and N-acetyltryptophan [43]. De-nitrosation of NANT can be mediated by XO through superoxidation [44]. So the significant reversion of serum N-acetyltryptophan may be related to the activation of uric acid and XO.

### Induced amino acid metabolism perturbation

In the oxalate group, significant changes in concentrations were observed for several amino acids, including the increase in aspartic acid and the decrease in tryptophan, tyrosine, isoleucine, and valine. Indolelactic acid is a metabolite of tryptophan. The elevation of serum indolelactic acid implies that the decrease in tryptophan may be caused by its accelerated metabolism, which in accordance with a previous metabolomics study of hydroxy-l-proline-induced CaOx nephrolithiasis in rats [45].

The therapeutic effect of FFJQC is correlated with the decrease in aspartic acids. The presence of urinary proteins helps in the destruction of CaOx crystals formed in the urinary tract and the prevention of kidney stones [46]. Strongest effects on crystallization were observed for polypeptides that are either highly hydrophilic and highly carboxylated (poly-l-aspartic acid, poly-l-glutamic acid), which can adsorb to CaOx monohydrate [47]. OPN is a prototype of the aspartic acid-rich proteins. Accumulating evidence suggests it is part of the body’s defense mechanism in urine against pathological CaOx crystallization [48], which shows different functions of OPN from it in the renal tissue. As being shown in a meta-analysis, in addition to urine, significantly lower OPN levels were detected also in the serum of urolithiasis patients as compared with the normal controls [49]. The decrease in serum aspartic acid in the FFJQC groups may imply the expression of serum OPN. The elevated OPN in serum may have a protective effect on the CaOx injured mice.

### Induced membrane lipids metabolism perturbation

Numerous studies have characterized the cytotoxicity of oxalate as plasma membrane damage and organelle injury [50]. In this study, phospholipids including phosphatidylcholine (PC), phosphatidylethanolamine (PE), phosphatidylserine (PS), and sphingolipids as the main components of biological membranes showed a significant increasing trend in the oxalate group, which may be related to the membrane toxicity of oxalate. Sphinganine is one kind of these metabolites. The down-regulated sphinganine (induced by FFJQC) may suggest that FFJQC may have protective effects on cell membranes.

Arachidonic acid (AA) is a polyunsaturated fatty acid produced from the phospholipids (especially PE, PC, and phosphatidylinositides) of cell membranes. AA is released with lysophospholipids such as lysophosphatidylcholine (LysoPC) from phospholipids when the cells are injured. This metabolomic pathway is mediated by phospholipases (e.g. phospholipases A2). In this experiment, the significant changes in the serum AA and their metabolic pathways in the oxalate group may be related to this process. In the present study, the model mice are injected with glyoxylate, a substrate for oxalate synthesis by glycolate oxidase [51]. Oxalate can trigger a spectrum of responses in renal cells in two ways including alterations in membrane surface properties that promote crystal attachment [52] and alterations in cell viability that provide debris for crystal nucleation [53]. Activation of cytosolic PLA2 appears to play an important role in oxalate actions, triggering a signaling cascade that generates several lipid mediators (AA; LysoPC; ceramide) that act on key intracellular targets (mitochondria, nucleus) [54]. Once being released by PLA2, free AA can play a direct role as a signal, but also can become a precursor that is metabolized by various enzymes to a wide range of biologically and clinically important eicosanoids. As being observed in the oxalate group, multiple metabolites of AA including oxoeicosatetraenoic acids, hydroxyeicosatetraenoic acids, hydroperoxyeicosatetraenoic acids, epoxyeicosatrienoic acids, and prostaglandins (PGs) were changed significantly.

Amongst the metabolites of AA, PGs are produced by cyclooxygenase (COX) following a sequential oxidation of AA. PGs are lipid mediators being involved in a variety of physiological and pathophysiological processes in the kidney, including renal hemodynamics, body water and sodium balance, and the inflammatory injury characteristic in multiple renal diseases [55]. The PG system is activated in response to obstructive nephropathy. Several studies have demonstrated that ureteral obstruction is associated with a marked induction of COX-2, and increased PGs and thromboxane synthesis [56]. Increased PGE2 secretion, as well as morphological alterations and necrotic cell death, was induced by CaOx on renal tubular cells in culture. Since previous studies were limited to environment of the kidney tissue, it is hard to say in our study whether the reversion of serum PGF2a, one of bioactive prostanoids followed by the production of PGE2, have the similar significance as being suggested.

### Induced energy perturbation

The elevation of serum free fatty acids and the activation of citrate cycle metabolism in the oxalate group may be related to the lipid mobilization so as to maintain energy homeostasis during injury. The accumulation of massive serum acylcarnitines including linoleylcarnitine, palmitoylcarnitine, stearoylcarnitine, hexadecadienoylcarnitine, octadecenylcarnitine, dodecanoylcarnitine, tetradecanoylcarnitine observed in the oxalate group may imply a mitochondrial injury caused by CaOx crystal. Acylcarnitines are the product of enzymatic esterification of carnitine, a water-soluble molecule vital for the transportation and oxidation of fatty acids in the mitochondria in healthy individuals. However, acylcarnitines especially with longer acyl chain length found in the plasma may suggest incomplete fatty acid β-oxidation and problems of mitochondrial energy metabolism [57]. A total of six differentially expressed proteins participated in the regulation of mitochondrial energy metabolism have been identified in neonatal rat kidney after partial unilateral ureteral obstruction [58]. Due to mitochondrial dysfunction, the induction of oxidative stress is induced and the acylcarnitines have been demonstrated, *in vitro*, to activate the inflammatory pathways [59].

In addition, lack of carnitine in serum has been observed in a previous serum metabolomic study of l-proline-induced CaOx nephrolithiasis in rats [45], which may be correlated to the mitochondrial dysfunction. Carnitine deficiency was indicated as a risk factor during cyclophosphamide-induced nephrotoxicity [60]. Carnitine has been demonstrated to protect renal tubular cells against CaOx monohydrate crystals adhesion through preventing cells from dedifferentiation [61]. Fatty acid β-oxidation is controlled by carnitine palmitoyltransferase 1. Stimulation of this enzyme has been found to improve renal function and slow down the damage of kidney tissue after ischemia–reperfusion [62]. Thus, we reason that the FFJQC plays an important role on the improvement of fatty acid β-oxidation and carnitine balance for crystal injured mice.

## Conclusion

In summary, the therapeutic benefits of FFJQC on oxalate crystal-injured kidney were observed by the decreased crystal disposition in a mice model. The expression of OPN and CDK44 were down-regulated, while calbindin-D28k were up-regulated by the treatment of FFJQC. In addition, the plasma metabolic profile of the injured mice was restored to the normal range after the treatment. Based on pathway analysis of the 81 identified metabolite biomarker candidates, the protective effect of FFJQC on crystaline nephropathy may be associated with the regulation of multiple metabolic pathways including purine metabolism, amino acid metabolism, membrane lipids metabolism, and energy metabolism.

## Supporting information

**Supplementary Figure 1:** 

**Supplementary Figure 2:** 

**Supplementary Figure 3:** 

**Table S1:** Potential biomarkers in FFJQC treatment for glyoxylate-induced crystal kidney injury, and their metabolic pathways.

## Abbreviations

AA: arachidonic acid
BUN: blood urea nitrogen
CaOx: calcium oxalate
CAT: catalase
CD44: cluster of differentiation 44
COX: cyclooxygenase
FFJQC: Fu-Fang-Jin-Qian-Chao
GSH: glutathion
HE: Hematoxylin–Eosin
i.g.: intragastrically
i.p.: intraperitoneally
LysoPC: lysophosphatidylcholine
MDA: malondialdehyde
NANT: N-Acetyl-N-nitroso-tryptophan
NIH: National Institutes of Health
OPN: osteopontin
PC: phosphatidylcholine
PCA: principal component analysis
PE: phosphatidylethanolamine
PG: prostaglandin
PLS-DA: partial least squares discriminant analysis
ROS: reactive oxygen species
Scr: serum creatinine
SOD: superoxide dismutase
XO: xanthine oxidase
