# Supplementary material for: Metabolomic analysis reveals a protective effect of Fu-Fang-Jin-Qian-Chao herbal granules on oxalate-induced kidney injury
Source: Biosci Rep. 2019 Feb 22;39(2):BSR20181833. doi: 10.1042/BSR20181833 (PMC6386768; doi:10.1042/BSR20181833)
Supplement: Supplementary file 1 [file bsr-39-bsr20181833_Supp1.pdf]

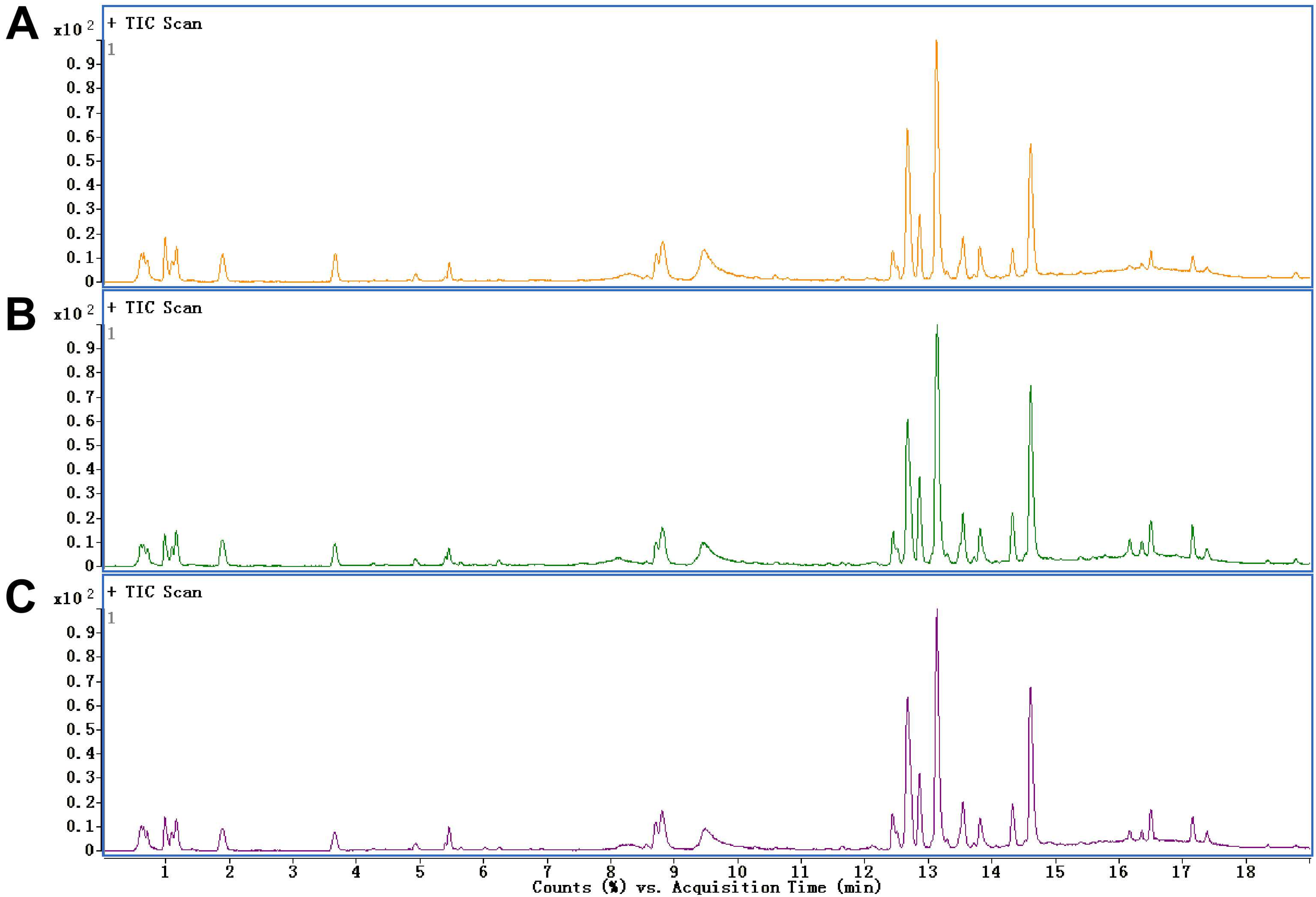

A
B
C
+ TIC Scan
x10 2
1
0.9
0.8
0.7
0.6
0.5
0.4
0.3
0.2
0.1
0
1 2 3 4 5 6 7 8 9 10 11 12 13 14 15 16 17 18
Counts (%) vs. Acquisition Time (min)

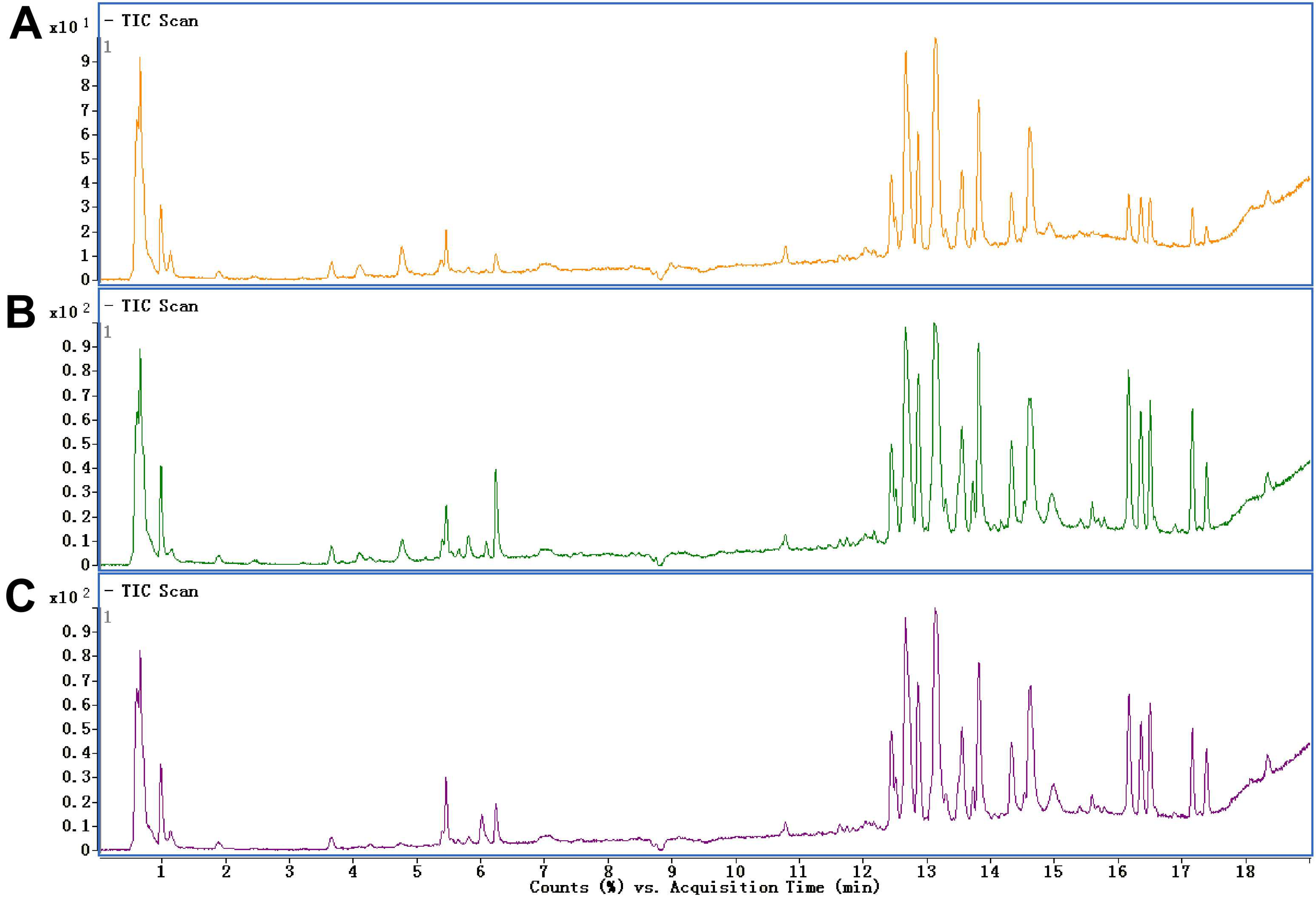

A
x10 1
- TIC Scan
B
x10 2
- TIC Scan
C
x10 2
- TIC Scan
Counts (%) vs. Acquisition Time (min)

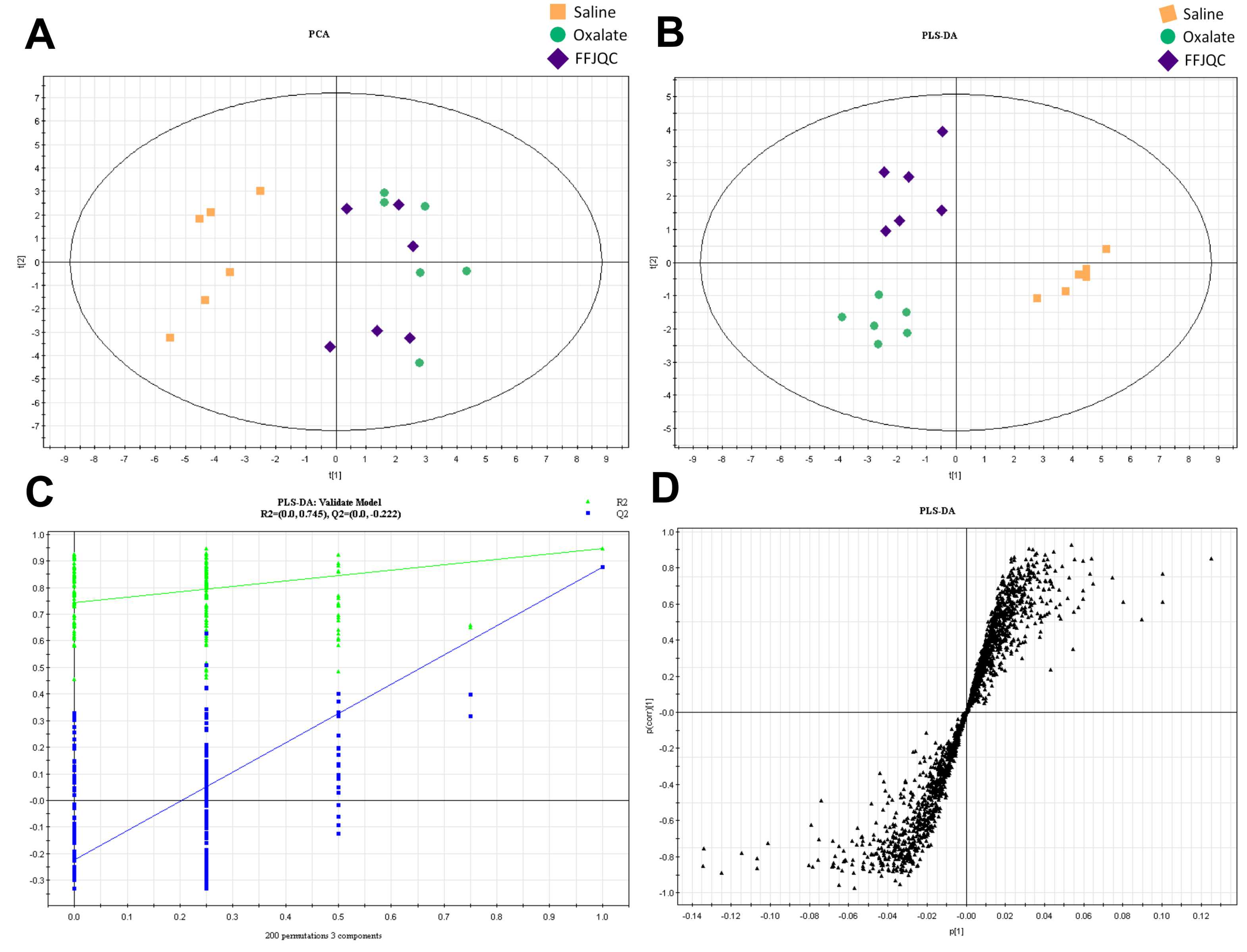
A
PCA
Saline
Oxalate
FFJQC
t[2]
t[1]
B
PLS-DA
Saline
Oxalate
FFJQC
t[2]
t[1]
C
PLS-DA: Validate Model
R2=(0.0, 0.745), Q2=(0.0, -0.222)
R2
Q2
200 permutations 3 components
D
PLS-DA
p(corr)[1]
p[1]

Table S1. Potential biomarkers in FFJQC treatment for glyoxylate-induced crystal kidney injury, and their metabolic pathways.

No.	RT(min)	m/z	Ion	Formula	Identification	Oxalate /Saline		FFJQC/ Oxalate		Related pathway
						P value	Fold change	P value	Fold change	
1	0.65	179.057	[M-H]$^-$	C6H12O6	D-Glucose	0.005	0.72	1.000	1.02	Glycolysis
2	0.67	140.068	[M+Na]$^+$	C5H11NO2	L-Valine	0.003	0.53	0.946	1.22	Valine, leucine and isoleucine degradation
3	0.67	162.112	[M+H]$^+$	C7H15NO3	L-Carnitine	<0.001	0.41	1.000	1.15	Bile secretion
4	0.68	132.029	[M-H]-	C4H7NO4	L-Aspartic acid	0.016	1.44	0.010	0.68	Alanine, aspartate and glutamate metabolism
5	0.70	157.036	[M-H]-	C4H6N4O3	Allantoin	0.004	1.48	0.018	0.73	Purine metabolism
6	0.99	135.031	[M-H]-	C5H4N4O	Hypoxanthine	<0.001	0.16	0.002	4.84	Purine metabolism
7		137.046	[M+H]$^+$							
8	0.99	153.041	[M+H]$^+$	C5H4N4O2	Xanthine	0.030	0.42	0.003	2.92	Purine metabolism
	0.99	169.036	[M+H]$^+$	C5H4N4O3	Uric acid	<0.001	3.72	0.009	0.54	Purine metabolism

9	0.99	381.03	[M-H]-	C16H14O9S	hesperetin 3'-O-sulfate	<0.001	2.30	0.092	0.79	
10	0.99	151.023	$[M+FA-H]^-$	C3H6O4	Glyceric acid	<0.001	0.27	0.002	3.02	Glycerolipid metabolism
11	1.00	173.01	[M-H]-	C6H6O6	cis-Aconitic acid	0.003	1.36	0.325	0.89	Citrate cycle
12	1.04	180.067	[M-H]-	C9H11NO3	L-Tyrosine	<0.001	0.52	0.243	1.30	Phenylalanine, tyrosine and tryptophan biosynthesis
13	1.07	129.02	[M-H]-	C5H6O4	Glutaconic acid	0.001	1.45	0.971	0.93	Fatty acid metabolism
14	1.08	189.041	[M-H]-	C7H10O6	3-Dehydroquinate	0.023	1.34	0.045	0.77	Phenylalanine, tyrosine and tryptophan biosynthesis
15	1.10	242.096	[M-H]-	C10H17N3O2S	Biotin amide	<0.001	0.43	1.000	1.04	
16	1.11	115.004	[M-H]-	C4H4O4	Fumaric acid	0.010	1.68	1.000	0.68	Citrate cycle
17	1.13	130.087	[M-H]-	C6H13NO2	L-Isoleucine	<0.001	0.50	1.000	1.05	Valine, leucine and isoleucine degradation
18	1.13	117.019	[M-H]-	C4H6O4	Succinic acid	0.040	1.47	0.098	0.73	Citrate cycle
19	1.13	267.075	[M-H]-	C10H12N4O5	Inosine	<0.001	0.03	0.008	21.04	Purine metabolism
20		269.088	$[M+H]^+$							
21	1.14	352.053	$[M+FA-H]^-$	C9H14N3O7P	dCMP	<0.001	0.23	0.024	2.98	Pyrimidine metabolism

	1.18	229.068	[M+FA-H]$^-$	C5H15NO4P	Phosphorylcholine	<0.001	7.17	0.305	0.89	Glycerophospholipid metabolism
22	3.66	203.084	[M-H]-	C11H12N2O2	L-Tryptophan	0.004	0.66	1.000	1.06	Phenylalanine, tyrosine and tryptophan biosynthesis
23	3.67	261.042	[M-H]-	C6H15O9P	Sorbitol-6-phosphate	<0.001	0.42	1.000	1.06	Fructose and mannose metabolism
24	4.23	228.088	[M+FA-H]$^-$	C9H13NO3	Epinephrine	<0.001	3.24	0.271	0.86	Tyrosine metabolism
25	5.81	187.008	[M-H]-	C7H8O4S	p-Cresol sulfate	0.048	1.89	0.017	0.42	Degradation of aromatic compounds
26	6.27	206.081	[M+H]$^+$	C11H11NO3	Indolelactic acid	0.004	1.58	0.007	0.66	Tryptophan metabolism
27		204.067	[M-H]-							
28	6.33	247.107	[M+H]$^+$	C13H14N2O3	N-acetyltryptophan	0.017	1.71	0.034	0.63	Tryptophan metabolism
		245.094	[M-H]-							
29	7.35	199.098	[M-H]-	C10H16O4	cis-4-Decenedioic acid	<0.001	5.58	0.110	0.65	Oxidation of fatty acids
	7.55	159.102	[M-H]-	C8H16O3	Hydroxyoctanoic acid	<0.001	2.81	1.000	0.89	Fatty acid metabolism
30	7.58	201.114	[M-H]-	C10H18O4	Sebacic acid	0.006	6.81	0.410	0.64	Fatty acid metabolism
31	9.19	185.117	[M-H]-	C10H18O3	3-Oxodecanoic acid	0.004	2.51	0.029	0.54	Pyrimidine metabolism
32	9.76	187.133	[M-H]-	C10H20O3	3-Hydroxycapric acid	<0.001	2.93	1.000	0.92	Oxidation of fatty acids

33	10.02	353.235	[M-H]-	C20H34O5	11b-PGF2a	0.004	1.96	0.401	0.80	Arachidonic acid metabolism
34	10.33	344.28	$[M+H]^+$	C19H37NO4	Dodecanoylcarnitine	<0.001	4.11	0.658	0.81	Fatty acid metabolism
35	10.65	353.235	[M-H]-	C20H34O5	Prostaglandin F2a	<0.001	3.95	0.015	0.61	Arachidonic acid metabolism
36	10.87	370.295	$[M+H]^+$	C21H39NO4	cis-5-Tetradecenoylcarnitine	<0.001	6.76	1.000	0.87	Fatty acid metabolism
37	11.20	396.311	$[M+H]^+$	C23H41NO4	9,12-Hexadecadienoylcarnitine	<0.001	9.40	0.514	0.78	Fatty acid metabolism
38	11.44	372.311	$[M+H]^+$	C21H41NO4	Tetradecanoylcarnitine	<0.001	4.72	0.366	0.75	Fatty acid metabolism
39	11.52	302.305	$[M+H]^+$	C18H39NO2	Sphinganine	0.029	1.67	0.016	0.56	Sphingolipid metabolism
40	11.65	215.166	[M-H]-	C12H24O3	3-Hydroxydodecanoic acid	<0.001	2.93	1.000	0.98	Fatty acid metabolism
41	11.79	398.326	$[M+H]^+$	C23H43NO4	trans-Hexadec-2-enoyl carnitine	<0.001	6.53	0.959	0.83	Fatty acid metabolism
42	11.94	586.316	$[M+FA-H]^-$	C28H48NO7P	LysoPC(20:5(5Z,8Z,11Z,14Z,17Z))	0.023	0.42	0.287	1.79	Glycerophospholipid metabolism
43	12.12	424.342	$[M+H]^+$	C25H45NO4	Linoleyl carnitine	0.001	4.00	1.000	0.89	Fatty acid metabolism
44	12.43	482.324	$[M+H]^+$	C23H48NO7P	LysoPE(0:0/18:0)	0.049	1.41	1.000	0.92	Glycerophospholipid metabolism
45	12.48	400.342	$[M+H]^+$	C23H45NO4	L-Palmitoylcarnitine	0.001	2.94	0.556	0.81	Fatty acid metabolism

46	12.54	526.293	$[M+H]^+$	C27H44NO7P	LysoPE(22:6/0:0)	0.022	3.22	0.331	0.62	Glycerophospholipid metabolism
47	12.71	241.182	[M-H]-	C14H26O3	3-Oxotetradecanoic acid	<0.001	6.67	1.000	0.97	Fatty acid metabolism
48	12.75	426.358	$[M+H]^+$	C25H47NO4	Octadecenylcarnitine	<0.001	4.01	0.581	0.82	Fatty acid metabolism
49	12.79	335.224	[M-H]-	C20H32O4	Leukotriene B4	0.046	1.55	1.000	0.96	Arachidonic acid metabolism
50	12.99	526.295	[M-H]-	C27H46NO7P	LysoPE(22:5/0:0)	0.001	1.66	0.110	0.81	Glycerophospholipid metabolism
51	13.06	454.293	$[M+H]^+$	C21H44NO7P	LysoPE(0:0/16:0)	0.019	1.59	1.000	0.89	Glycerophospholipid metabolism
52	13.07	317.214	[M-H]-	C20H30O3	5-Oxoeicosatetraenoic acid	0.001	2.44	1.000	0.91	Arachidonic acid metabolism
53	13.10	812.546	[M-H]-	C44H80NO10P	PS(20:3/18:0)	0.001	2.35	1.000	0.88	Glycerophospholipid metabolism
54	13.40	243.197	[M-H]-	C14H28O3	2-Hydroxymyristic acid	<0.001	2.83	1.000	0.95	
55	13.46	428.373	$[M+H]^+$	C25H49NO4	Stearoylcarnitine	0.022	1.96	1.000	0.87	Fatty acid metabolism
56	13.54	522.357	$[M+H]^+$	C26H52NO7P	LysoPC(18:1(9Z))	0.030	1.49	1.000	0.97	Glycerophospholipid metabolism
57	13.72	379.206	$[M+FA-H]^-$	C20H30O4	Delta-12-Prostaglandin J2	0.001	2.57	0.238	0.77	Arachidonic acid metabolism
58	13.72	864.578	[M-H]-	C48H84NO10P	PE(MonoMe(9,5)/DiMe(13,5))	<0.001	2.85	0.037	0.74	Glycerophospholipid metabolism
59	13.72	343.23	[M-H]-	C22H32O	4-Hydroxy-docosahexaenoic acid	0.001	2.69	0.517	0.82	Oxidation of fatty acids

60	13.77	616.363	[M-H]-	C32H51N5O7	Neuromedin N	0.008	0.63	1.000	1.14	
61	13.81	343.225	$[M+Na]^+$	C20H32O3	8,9-Epoxyeicosatrienoic acid	0.017	1.77	1.000	0.86	arachidonic acid
62	13.82	319.231	[M-H]-	C20H32O3	12-hydroxyeicosatetraenoic acid	0.013	1.65	1.000	0.93	Arachidonic acid metabolism
63	13.82	377.189	[M-H]-	C17H26N6O4	Pentosidine	0.015	1.50	1.000	0.91	
64	13.82	301.219	[M-H]-	C20H30O2	Eicosapentaenoic acid	0.006	1.69	1.000	0.93	Biosynthesis of unsaturated fatty acids
65	13.87	510.356	$[M+H]^+$	C25H52NO7P	LysoPC(17:0)	0.018	1.61	0.926	0.87	Glycerophospholipid metabolism
66	13.91	508.376	$[M+H]^+$	C26H54NO6P	LysoPC(P-18:0)	0.025	1.78	0.678	0.82	Glycerophospholipid metabolism
67	14.08	317.214	[M-H]-	C20H30O3	12-Oxoeicosatetraenoic acid	0.042	1.91	1.000	0.86	Arachidonic acid metabolism
68	14.17	305.247	$[M+H]^+$	C20H32O2	Arachidonic acid	<0.001	3.45	0.257	0.75	Arachidonic acid metabolism
69	14.17	321.245	[M-H]-	C20H34O3	15(S)-Hydroxyeicosatrienoic acid	0.001	2.88	0.394	0.78	Arachidonic acid metabolism
70	14.61	1047.74	$[M+Na]^+$	C69H100O6	TG(22:5/22:6)	0.007	1.97	1.000	0.87	Fatty acid metabolism
71	15.66	271.229	[M-H]-	C16H32O3	3-hydroxyhexadecanoic	<0.001	2.49	0.048	0.87	Fatty acid metabolism

					acid					
72	16.07	719.535	[M-H]-	C37H75N2O6P	SM(d18:1/14:0)	0.005	0.44	1.000	1.14	Sphingolipid metabolism
73	16.11	253.218	[M-H]-	C16H30O2	Palmitoleic acid	<0.001	3.94	0.480	0.81	Fatty acid metabolism
74	16.17	363.211	[M-H]-	C21H32O5	Tetrahydrocortisone	0.004	3.04	1.000	0.91	Steroid hormone biosynthesis
75	16.17	329.248	$[M+H]^+$	C22H32O2	Docosahexaenoic acid	0.001	2.06	1.000	0.91	Biosynthesis of unsaturated fatty acids
76	16.50	398.134	[M-H]-	C15H23N6O5S	S-Adenosylmethionine	<0.001	0.39	1.000	1.01	Cysteine and methionine metabolism
77	16.51	279.235	[M-H]-	C18H32O2	linoleic acid	0.004	1.78	0.482	0.89	Fatty acid metabolism
78	17.16	255.235	[M-H]-	C16H32O	Palmitic acid	<0.001	2.76	1.000	0.98	Fatty acid metabolism
79	17.39	281.251	[M-H]-	C18H34O2	Oleic acid	<0.001	2.64	0.211	0.81	Fatty acid metabolism
80	18.35	283.265	[M-H]-	C18H36O2	Stearic acid	0.004	1.63	1.000	0.97	Fatty acid metabolism
81	18.48	309.281	[M-H]-	C20H38O2	Eicosenoic acid	0.009	1.78	1.000	0.88	Biosynthesis of unsaturated fatty acids

FFJQC, Fu-Fang-Jin-Qian-Chao; SM, Sphingomyelin; PE, phosphatidyl ethanolamine; PC, phosphatidylcholine; LysoPE, lysophosphatidyl ethanolamine; LysoPC, lysophosphatidylcholine; dCMP stands for deoxycytidine monophosphate
